# Growing disparity in the prevalence of chronic obstructive pulmonary disease between people with and without disabilities: a Korean nationwide serial cross-sectional study

**DOI:** 10.1038/s41598-023-39319-8

**Published:** 2023-08-14

**Authors:** Jinsoo Min, Jong Eun Park, So Young Kim, Yeon Yong Kim, Jong Hyock Park

**Affiliations:** 1 Division of Pulmonary and Critical Care Medicine, Department of Internal Medicine, Seoul St. Mary’s Hospital, College of Medicine, The Catholic University of Korea, Seoul, South Korea; 2 Institute of Health & Science Convergence, Chungbuk National University, Cheongju, South Korea; 3 Department of Public Health and Preventive Medicine, Chungbuk National University Hospital, Cheongju, South Korea; 4 Big Data Steering Department, National Health Insurance Service, Wonju, South Korea; 5 Drug Evaluation Department, National Institute of Food and Drug Safety Evaluation, Cheongju, South Korea; 6 Department of Medicine, College of Medicine, Chungbuk National University, Cheongju, South Korea

**Keywords:** Respiratory tract diseases, Public health, Risk factors, Epidemiology

## Abstract

Few studies have examined the association between disability and chronic obstructive pulmonary disease (COPD). We compared the trends in the annual COPD prevalence between people with and without disabilities, and examined the association between disability and COPD. We linked the National Health Information Database (2008–2017) with the National Disability Registration Database, which includes more than 2 million people with disabilities every year. In the 2017 dataset, people with disabilities had a higher prevalence of COPD than those without disabilities (30.6% vs. 12.5%, *P* < 0.001). The age-standardized prevalence rate of COPD among people without disabilities increased from 4.2 in 2008 to 10.9% in 2017 (change of 6.7%), whereas that among those with disabilities increased from 7.0 to 17.1% (change of 10.1%). In multivariate analysis, compared to people without disabilities, those with disabilities had a higher probability of having COPD (adjusted odds ratio, 1.42; 95% confidence interval 1.42–1.43). The results of subgroup analysis by disability characteristics suggested that disabilities due to failure of an organ, such as the kidney, lung, heart, or liver, and severe disabilities were particularly vulnerable to COPD. In conclusion, people with disabilities are more likely to have COPD compared to people without disabilities. Further longitudinal studies that examine cause-and-effect relationship between disability and COPD are needed to clarify this relationship and to further investigate any potential negative effects associated with the coexistence of these conditions.

## Introduction

Chronic obstructive pulmonary disease (COPD) is a major global health issue and the third leading cause of death worldwide^1^. With a worldwide prevalence of 10.1%, COPD afflicts many people in both low- and high-income countries^2^. As COPD progresses, affected people find it more difficult to do normal daily activities, and a considerable financial burden is imposed by limitations in workplace and home productivity, and by the costs of medical treatment. However, COPD is greatly underdiagnosed and often not identified until late in its course^3^.

People with disabilities, who tend to have poor health and additional healthcare needs due to underlying health problems^4^, may be more vulnerable to chronic diseases. The range of disabilities is extremely diverse, and includes physical, sensory, mental, intellectual, and communication impairments. Approximately 15% of the world population lives with some form of disability^5^. Even in South Korea, which defines disability in a strict manner, the reported prevalence rate is about 5.1%^6^, and the number of people with disabilities is expected to continue to increase worldwide due to an aging population and rapid increase in the prevalence of chronic diseases.

People with disabilities receive scant attention in terms of public health, although recent efforts have increased awareness of health disparities in relation to this population^7,8^. People with disabilities have multiple risk factors that increase their chances of developing many chronic diseases, such as physical inactivity and high rates of obesity and smoking^9,10^. Many people with disabilities have a lower educational level than people without disabilities, and are less economically active and more likely to experience poverty; this makes it more difficult for them to access healthcare services. It is therefore important to better understand the chronic diseases commonly suffered by people with disabilities, and to devise appropriate public health interventions for persons with disabilities who also have comorbidities.

Adults with COPD have a tenfold higher risk of disability than the general population^11,12^; however, there is little research regarding how many people with disability suffer from respiratory conditions. Although some studies have evaluated the association of COPD with frailty and pre-frailty^13^, the prevalence of chronic lung diseases, such as COPD, among people with disabilities is not known. We hypothesized that people with disabilities are at greater risk of developing COPD, because disability and COPD share several risk factors such as physical inactivity, smoking, obesity, arrested lung development, and increased exposure to occupational agents. The purpose of this study was to compare annual trends of COPD prevalence between people with and without disabilities, and to clarify the association between disability and COPD by analyzing medical big data generated by the Korean national health insurance system for national health insurance subscribers and Medical Aid recipients.

## Methods

### Study design and population

We conducted a nationwide serial cross-sectional study using national registry databases. We linked the National Disability Registration Database with the National Health Information Database (NHID) of the Republic of Korea. The NHID is a public database containing information on health care utilization, health screening, sociodemographics, and mortality for the whole Korean population; it is maintained by the Korean National Health Insurance Service (NHIS)^14^.

We extracted information on disability type and severity from the National Disability Registration Database for people with disabilities between 2008 and 2017. The Korean government established a national disability registration system in 1988 to provide welfare benefits to people with 15 types of disabilities, and defined six levels of disability severity^15^. Disability registration requires submission of validated documentation, including appraised results of disability diagnosis by a specialist physician in the corresponding field according to detailed criteria. The database covered 93.8% of the total population with disabilities in 2011. Based on personal identification numbers, disability types and severity were linked with the variables of interest from the NHID.

### Definition of COPD

Because the NHID does not include clinical data pertaining to the diagnosis and treatment of diseases, such as the spirometry data allowing for the diagnosis of COPD, we identified COPD patients based on diagnostic codes. Similar to previous studies, COPD patients were identified herein based on the International Classification of Disease-Tenth Revision (ICD-10) diagnostic codes for COPD (ICD-10: J41–J44), completion at least two treatment courses, or at least one hospitalization during the current or preceding year.

### Independent variables

We collected data on factors that may influence the prevalence of COPD, such as sex, age, household income level, place of residence, and comorbidities. Household income was categorized based on the NHIS insurance contribution, which is in turn based on the monthly wage for employees (employee-insured) and metrics of household wealth (e.g., income, property, and car ownership) for the self-employed (self-employed-insured). The categories are as follows Medical Aid recipient, and 1st (lowest 25%), 2nd, 3rd, and 4th (highest 25%) insurance contribution quartiles. Residential area was classified as metropolitan, urban, or rural, based on the ZIP code. The cumulative burden of comorbidities for each subject was captured by the Charlson comorbidity index (CCI). Subjects were classified into four groups according to the CCI (0, 1–2, 3–4, and ≥ 5 [most severe comorbidities]).

### Statistical analyses

Descriptive statistics were generated for general participant characteristics. We calculated the prevalence of COPD for each year between 2008 and 2017. Annual COPD prevalence rates were compared between people with and without disabilities, stratified by sex. The age-standardized prevalence rate of COPD was also calculated, using the direct standardization method; the mid-year Korean population of 2005 was taken as the standard population. To examine the association between disability and COPD, we constructed a multivariable logistic regression model, adjusted for age, sex, income level, residence, and CCI, based on two datasets (2008 and 2017). All analyses were performed using SAS software (ver. 9.4; SAS Institute, Cary, NC, USA). Two-sided P-values of 0.05 were considered significant. In a subgroup analysis, we calculated the age-standardized prevalence rates of COPD according to disability severity and type, and assessed trends therein, based on two datasets (2008 and 2017). A multivariable logistic regression model stratified by disability type and severity was also constructed. For subgroup analysis, we excluded people with respiratory disability. We further stratified by sex and age and conducted the multivariable logistic regression analysis adjusted for other variables, based on the most recent dataset (2017). When divided into two groups by age, we set the standard for age as 60 years, the average age of people with disabilities.

The national disability registration data distinguishes 15 types of disability. Disability severity is graded from 1 (*very severe*) to 6 (*very mild*) based on functional and clinical impairments, as determined by a medical specialist. In the present study, disability severity was classified as severe (grade 1–3) or mild (grade 4–6). Additionally, the 15 disability types were reclassified into 9 categories: physical disability, brain injury, facial disability, visual disability, hearing and language disability, developmental disability (autism and intellectual disability), mental disability, internal organ disability (renal disease, heart disease, liver disease, ostomy, and epilepsy), and respiratory disability. Physical disability indicates any physical limitations or disabilities that inhibit physical function of one or more limbs due to amputation or other musculoskeletal or neuromuscular impairments. People with COPD, who required continuous oxygen therapy, were defined as having disability and classified into the respiratory disability, instead of the physical disability.

### Ethics approval

This study was approved by the International Review Board (IRB) of Chungbuk National University (IRB No. CBNU-202010-HRHR-0171). Because the database used in this study was based on routinely collected administrative and claims data, written informed consent was waived by the IRB of Chungbuk National University. Under Korea’s National Health Insurance Act, NHIS data can only be used for research purposes without the patient’s individual consent and were fully anonymized for all analyses. All works were performed in accordance with the Declaration of Helsinki.

## Results

### General characteristics of people with and without disabilities

The total number of people with disabilities enrolled in the health insurance system increased from 2,338,534 in 2008 to 2,627,365 in 2017 (Supplementary Tables 1, 2). The percentage of people with disabilities increased from 4.6% in 2008 to 5.0% in 2017.

The most recent dataset (2017) was analyzed to obtain the baseline characteristics of people with and without disabilities (Table 1). Of those with disabilities in the 2017 dataset, 38.4% had a severe disability, of which the most frequent was a physical disability (50.2%). The percentage of men was significantly higher in people with disabilities than those without disabilities (58.0% vs. 49.7%, *P* < 0.001). People with disabilities were also older (60.5 ± 18.0 vs. 40.0 ± 21.0), more likely to report a lower income and live in a rural area, and had more comorbidities (all *P* < 0.001).

**Table 1:** Characteristic of people with and without disabilities in the 2017 dataset.

	Total population (N = 52,712,239)	Without disability (N = 50,084,874)	With disability (N = 2,627,365)	*P* value
	N (Col%)	N (Col%)	N (Col%)	
Severity of disability				
Mild disability			1,618,856 (61.6)	
Severe disability			1,008,509 (38.4)	
Type of disability				
Physical disability			1,319,712 (50.2)	
Brain injury			258,610 (9.8)	
Facial disability			2,725 (0.1)	
Visual disability			259,423 (9.9)	
Hearing and language disability			327,694 (12.5)	
Developmental disability			226,281 (8.6)	
Mental disability			91,560 (3.5)	
Internal organ disability			128,565 (4.9)	
Respiratory problems			12,795 (0.5)	
Gender				
Male	26,390,827 (50.1)	24,866,269 (49.7)	1,524,558 (58.0)	< 0.001
Female	26,321,412 (49.9)	25,218,605 (50.4)	1,102,807 (42.0)	
Age, years				
Mean ± SD	41.0 ± 21.3	40.0 ± 21.0	60.5 ± 18.0	< 0.001
< 20	9,800,667 (18.6)	9,709,636 (19.4)	91,031 (3.5)	< 0.001
20–29	7,039,984 (13.4)	6,946,162 (13.9)	93,822 (3.6)	
30–39	7,634,258 (14.5)	7,490,900 (15.0)	143,358 (5.5)	
40–49	8,788,690 (16.7)	8,502,051 (17.0)	286,639 (10.9)	
50–59	8,583,409 (16.3)	8,072,451 (16.1)	510,958 (19.5)	
60–69	5,723,391 (10.9)	5,150,543 (10.3)	572,848 (21.8)	
70–79	3,423,916 (6.5)	2,845,710 (5.7)	578,206 (22.0)	
≥ 80	1,717,924 (3.3)	1,367,421 (2.7)	350,503 (13.3)	
Income level				< 0.001
First quartile^a^	10,654,182 (20.2)	9,717,705 (19.4)	936,477 (35.6)	
Second quartile	10,426,396 (19.8)	10,031,017 (20.0)	395,379 (15.1)	
Third quartile	13,156,971 (25.0)	12,641,694 (25.2)	515,277 (19.6)	
Fourth quartile	17,260,428 (32.7)	16,516.505 (33.0)	743,923 (28.3)	
Unknown	1,214,262 (2.3)	1,177,953 (2.4)	36,390 (1.4)	
Place of residence				< 0.001
Metropolitan	32,887,047 (62.4)	31,449,131 (62.8)	1,437,916 (54.7)	
Urban	15,279,473 (29.0)	14,460,602 (28.9)	818,871 (31.2)	
Rural	4,519,0894 (8.6)	4,148,524 (8.3)	370,565 (14.1)	
Unknown	26,630 (0.1)	26,617 (0.1)	13 (0.0)	
CCI				< 0.001
0	30,346,892 (57.6)	29,536,748 (59.0)	810,144 (30.8)	
1–2	17,325,153 (32.9)	16,363,369 (32.7)	961,784 (36.6)	
3–4	3,550,426 (6.7)	3,038,469 (6.1)	511,957 (19.5)	
≥ 5	1,489,768 (2.8)	1,146,288 (2.3)	343,480 (13.1)	

*SD* standard deviation, *CCI* Charlson cormorbidity index.

^a^Medical Aid beneficiaries were merged into the first quartile group.

### Trends and differences in COPD prevalence among people with and without disabilities

In the most recent dataset (2017), 7,085,279 participants had COPD (prevalence 13.4%; Table 2). COPD was more prevalent among people with than without disabilities (30.6% vs. 12.5%, *P* < 0.001), regardless of sex, age, income level, place of residence, or CCI.

**Table 2:** Characteristics and prevalence of chronic obstructive pulmonary disease among people with and without disabilities in the 2017 dataset.

	Total population		Without disability		With disability		*P* value^a^
	No. of COPD cases	Crude prevalence rate, %	No. of COPD cases	Crude prevalence rate, %	No. of COPD cases	Crude prevalence rate, %	
All	7,085,279	13.4	6,282,610	12.5	802,669	30.6	< 0.001
Gender							
Male	3,143,878	11.9	2,722,083	11.0	421,795	27.7	< 0.001
Female	3,941,401	15.0	3,560,527	14.1	380,874	34.5	
Age, years							
< 20	784,304	8.0	772,959	8.0	11,345	12.5	< 0.001
20–29	411,012	5.8	400,966	5.8	10,046	10.7	< 0.001
30–39	569,931	7.5	552,116	7.4	17,815	12.4	< 0.001
40–49	834,622	9.5	792,084	9.3	42,538	14.8	< 0.001
50–59	1,165,137	13.6	1,059,248	13.1	105,889	20.7	< 0.001
60–69	1,321,530	23.1	1,140,409	22.1	181,121	31.6	< 0.001
70–79	1,234,862	36.1	979,168	34.4	255,694	44.2	< 0.001
≥ 80	763,881	44.5	585,660	42.8	178,221	50.9	< 0.001
Income level							
First quartile^b^	1,670,531	15.7	1,387,165	14.3	283,366	30.3	< 0.001
Second quartile	1,235,043	11.8	1,128,946	11.3	106,097	26.8	< 0.001
Third quartile	1,616,669	12.3	1,469,615	11.6	147,054	28.5	< 0.001
Fourth quartile	2,440,555	14.1	2,186,012	13.2	254,543	34.2	< 0.001
Unknown	122,481	10.1	110,872	9.4	11,609	32.0	< 0.001
Place of residence							
Metropolitan	4,181,505	12.7	3,768,492	12.0	413,013	28.7	< 0.001
Urban	2,080,051	13.6	1,825,836	12.6	254,215	31.0	< 0.001
Rural	823,632	18.2	688,191	16.6	135,441	36.6	< 0.001
CCI							
0	1,977,841	6.5	1,880,477	6.4	97,364	12.0	< 0.001
1–2	3,180,268	18.4	2,887,401	17.7	292,867	30.5	< 0.001
3–4	1,214,817	34.2	993,505	32.7	221,312	43.2	< 0.001
≥ 5	712,353	47.8	521,227	45.5	191,126	55.6	< 0.001

*COPD* chronic obstructive pulmonary disease, *CCI* Charlson cormorbidity index.

^a^*P* values for comparison of prevalence by disability status within stratified participants according to various characteristics.

^b^Medical aid beneficiaries were merged into the first quartile group.

**Figure 1:**
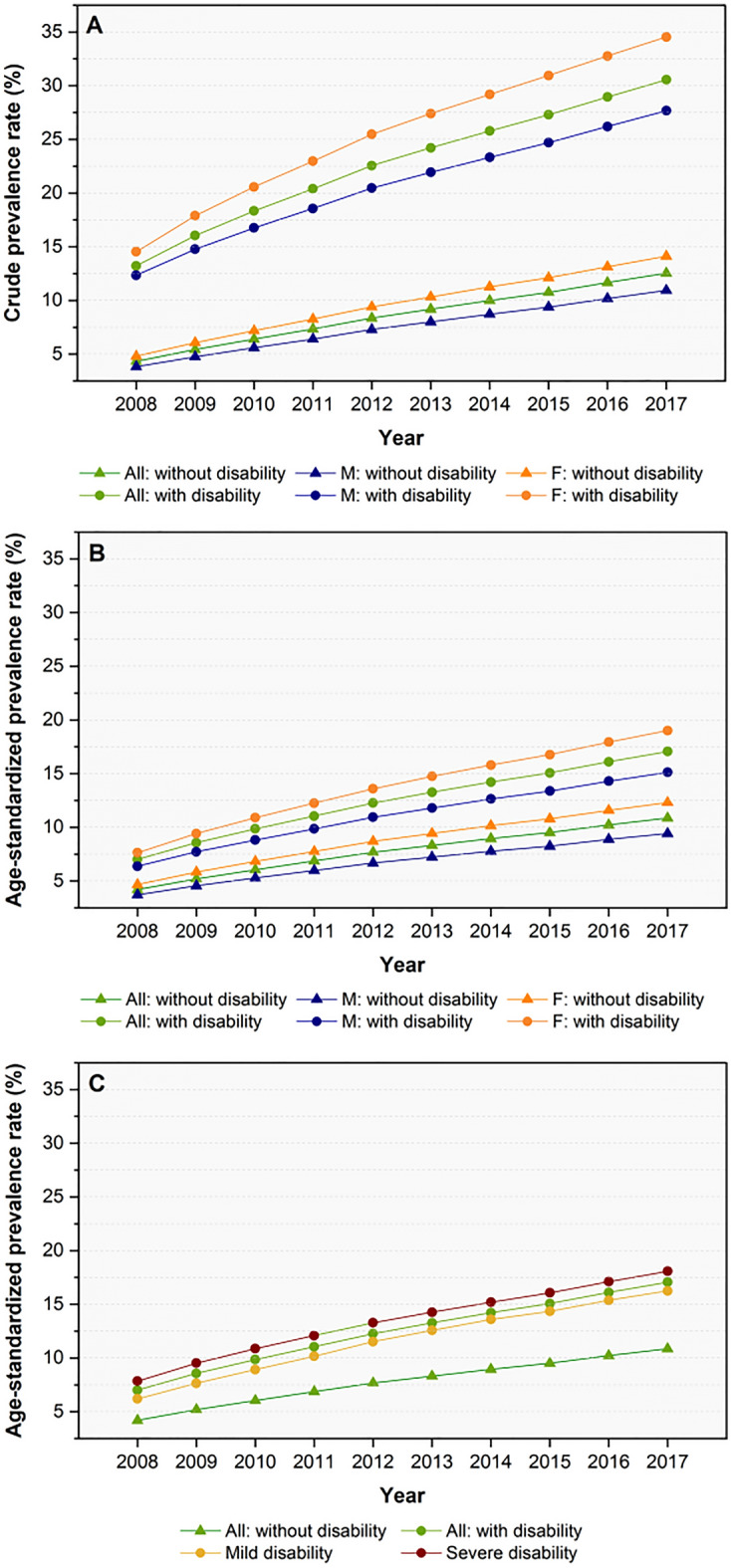
Trends in the prevalence rate of chronic obstructive pulmonary disease between 2008 and 2017 among people with and without disabilities. (**A**) Crude prevalence rate; (**B**) age-standardized prevalence rate; and (**C**) age-standardized prevalence rate stratified by disability severity.

From 2008 to 2017, the non-standardized and age-standardized prevalence rates of COPD increased in both groups with and without disabilities (Fig. 1A,B, Supplementary Table 3). However, COPD prevalence among people with disabilities was higher than among people without disabilities in all years. Women with disabilities had a higher prevalence of COPD than men with disabilities and all people without disabilities.

**Figure 2:**
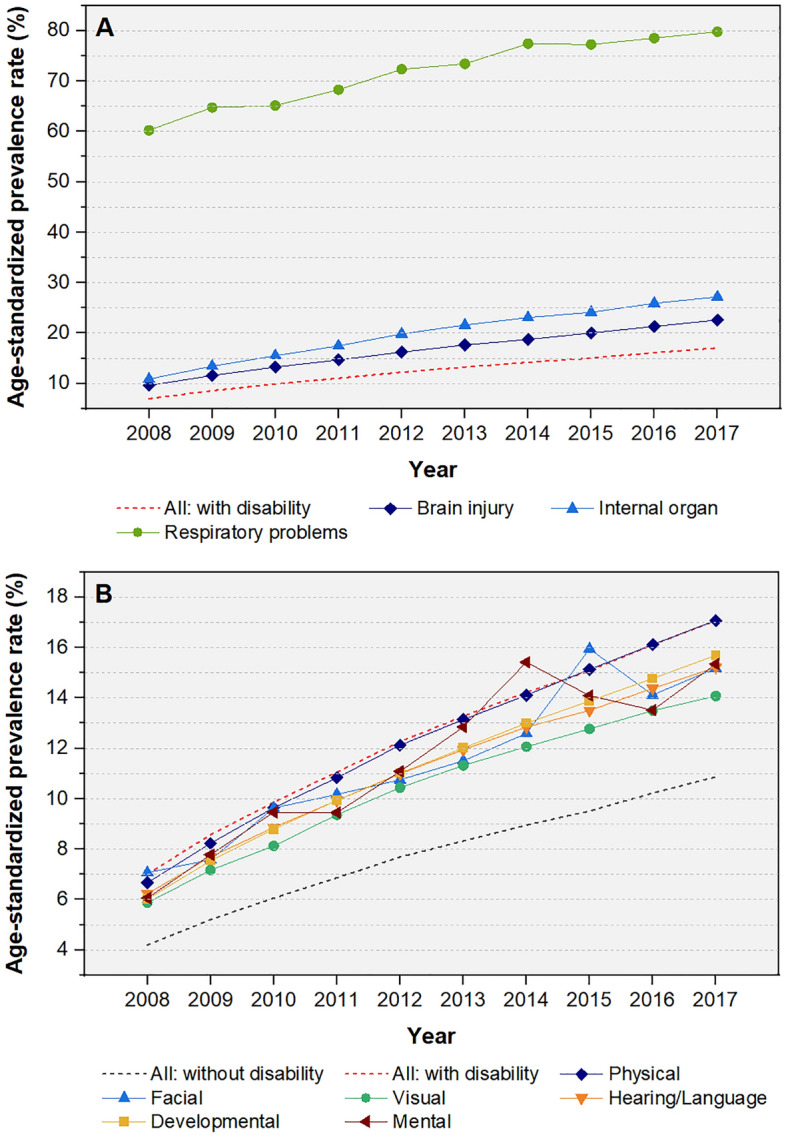
Age-standardized prevalence rate of chronic obstructive pulmonary disease between 2008 and 2017 stratified by type of disability. (**A**) Types of disabilities with a higher than average COPD prevalence among all disability subtypes. (**B**) Types of disabilities with at least an average COPD prevalence among all disability subtypes.

The age-standardized prevalence rates of COPD also increased over the study period in all disability severity (Fig. 1C) and type subgroups (Fig. 2A,B). People with disabilities due to failure of an organ, such as the kidney, lung, heart, or liver, had a higher prevalence of COPD than those with other types of disabilities. Also, the severe disability subgroup had a higher COPD prevalence rate than the mild disability subgroup.

The age-standardized prevalence rate of COPD among people without disabilities increased from 4.2 in 2008 to 10.9% in 2017 (change of 6.7%), whereas that among those with disabilities increased from 7.0 to 17.1% (change of 10.1%) (Table 3). Among the various types of disability, the highest COPD prevalence rate and largest increase therein were observed in people with respiratory disability (from 60.2 to 79.8%; change of 19.5%), internal organ disability (from 10.9 to 27.2%; change of 16.3%), and brain injury (from 9.6 to 22.6%; change of 13.0%). The lowest COPD prevalence rate and smallest increase were observed in people with visual (from 5.9 to 14.1%; change of 8.2%), facial (from 7.1 to 15.2%; change of 8.1%), and hearing and language (from 6.2 to 15.2%; change of 9.0%) disabilities.

**Table 3:** Prevalence of chronic obstructive pulmonary disease stratified by severity and type of disability in the 2008 and 2017 datasets.

	2008			2017			Absolute difference in prevalence of COPD (B vs. A)
	No. of COPD cases	Crude prevalence rate, %	(A) Age-standardized prevalence rate, %	No. of COPD cases	Crude prevalence rate, %	(B) Age-standardized prevalence rate, %	
Status of disability							
All							
Without disability	2,077,523	4.3	4.2	6,282,610	12.5	10.9	6.7
With disability	309,219	13.2	7.0	802,669	30.6	17.1	10.1
With disability (excluding respiratory disability)	295,920	12.7	6.8	790,671	30.2	16.9	10.1
Male							
Without disability	915,768	3.8	3.7	2,722,083	11.0	9.4	5.7
With disability	173,785	12.4	6.4	421,795	27.7	15.1	8.8
With disability (excluding respiratory disability)	163,351	11.7	6.1	412,802	27.3	15.0	8.9
Female							
Without disability	1,161,755	4.8	4.7	3,560,527	14.1	12.3	7.6
With disability	135,434	14.5	7.6	380,874	34.5	19.0	11.4
With disability (excluding respiratory disability)	132,569	14.3	7.5	377,869	34.4	18.9	11.4
Severity of disability							
Mild disability	172,676	12.9	6.2	513,711	31.7	16.2	10.1
Severe disability	136,543	13.7	7.9	288,958	28.7	18.1	10.2
Type of disability							
Physical disability	156,368	12.4	6.7	404,323	30.6	17.1	10.4
Brain injury	40,942	17.5	9.6	92,440	35.7	22.6	13.0
Facial disability	167	7.3	7.1	561	20.6	15.2	8.1
Visual disability	28,356	12.0	5.9	72,490	27.9	14.1	8.2
Hearing and language disability	40,397	16.5	6.2	121,010	36.9	15.2	9.0
Developmental disability	8047	4.8	6.0	33,025	14.6	15.7	9.7
Mental disability	5487	7.3	6.1	17,926	19.6	15.3	9.3
Internal organ disability	16,156	16.5	10.9	488,96	38.0	27.2	16.3
Respiratory problems	13,299	82.6	60.2	11,998	93.8	79.8	19.5

*COPD* chronic obstructive pulmonary disease.

### Disability characteristics associated with COPD prevalence

In multivariate logistic analyses controlling for sex, age, income level, place of residence, and CCI, the presence of a disability was associated with a higher prevalence of COPD (Table 4). People with disabilities had higher odds of COPD compared to those without disabilities (adjusted odds ratio [aOR] 1.42, 95% confidence interval [CI] 1.42–1.43). After stratifying by sex, the odds of COPD among people with disabilities were similar in both male (aOR 1.46, 95% CI 1.45–1.47) and female (aOR 1.38, 95% CI 1.37–1.38) (Table 5A). The odds of COPD among people with disabilities were also similar in both younger people < 60 years (aOR 1.50, 95% CI 1.50–1.51) and older people ≥ 60 years (aOR 1.28, 95% CI 1.28–1.29) (Table 5B).

**Table 4:** Association between disability and chronic obstructive pulmonary disease in the 2008 and 2017 datasets: results of multivariate logistic regression analysis.

	2008		2017	
	Crude OR (95% CI)	Adjusted OR (95% CI)^a^	Crude OR (95% CI)	Adjusted OR (95% CI)^a^
Status of disability				
With no disability	1.00	1.00	1.00	1.00
With disability	3.37 (3.36–3.38)	1.25 (1.25–1.26)	3.07 (3.06–3.08)	1.42 (1.42–1.43)
With disability (excluding respiratory disability)	3.23 (3.22–3.24)	1.20 (1.20–1.21)	3.02 (3.01–3.03)	1.40 (1.40–1.41)
Severity of disability (excluding respiratory disability)				
With no disability	1.00	1.00	1.00	1.00
Mild disability	3.26 (3.25–3.28)	1.22 (1.22–1.23)	2.69 (2.68–2.70)	1.40 (1.40–1.41)
Severe disability	3.19 (3.17–3.21)	1.17 (1.16–1.18)	3.24 (3.23–3.25)	1.40 (1.39–1.41)
Type of disability (excluding respiratory disability)				
With no disability	1.00	1.00	1.00	1.00
Physical disability	3.13 (3.11–3.14)	1.24 (1.23–1.25)	3.08 (3.07–3.09)	1.43 (1.42–1.43)
Brain injury	4.68 (4.63–4.73)	1.01 (1.00–1.02)	3.88 (3.85–3.91)	1.28 (1.27–1.29)
Facial disability	1.75 (1.49–2.04)	1.23 (1.04–1.45)	1.81 (1.65–1.98)	1.30 (1.18–1.44)
Visual disability	3.01 (2.97–3.05)	1.15 (1.13–1.16)	2.70 (2.68–2.73)	1.26 (1.25–1.28)
Hearing and language disability	4.37 (4.32–4.41)	1.46 (1.44–1.48)	4.08 (4.05–4.11)	1.60 (1.58–1.61)
Developmental disability	1.12 (1.09–1.14)	1.33 (1.30–1.37)	1.19 (1.18–1.21)	1.44 (1.42–1.45)
Mental disability	1.73 (1.68–1.78)	1.22 (1.18–1.25)	1.70 (1.67–1.73)	1.23 (1.21–1.25)
Internal organ disability	4.36 (4.29–4.44)	0.91 (0.90–0.93)	4.28 (4.23–4.33)	1.30 (1.29–1.32)

*OR* odds ratio, *CI* confidence interval, *CCI* Charlson cormorbidity index.

^a^Adjusted for age (continuous), sex, income level, place of residence, and Charlson cormorbidity index.

**Table 5:** Stratified analysis to assess association between disability and chronic obstructive pulmonary disease in the 2017 dataset; (A) stratified by sex, and (B) stratified by age.

(A) Sex	Crude OR (95% CI)	Adjusted OR (95% CI)^a^
Male		
Status of disability		
With no disability	1.00	1.00
With disability	3.11 (3.10–3.12)	1.46 (1.45–1.47)
With disability (excluding respiratory disability)	3.05 (3.04–3.06)	1.43 (1.42–1.44)
Severity of disability (excluding respiratory disability)		
With no disability	1.00	1.00
Mild disability	3.12 (3.11–3.14)	1.40 (1.39–1.41)
Severe disability	2.94 (2.92–2.95)	1.48 (1.47–1.49)
Type of disability (excluding respiratory disability)		
With no disability	1.00	1.00
Physical disability	2.85 (2.83–2.86)	1.40 (1.40–1.41)
Brain injury	4.32 (4.28–4.37)	1.35 (1.34–1.37)
Facial disability	1.81 (1.06–2.06)	1.31 (1.15–1.50)
Visual disability	2.80 (2.76–2.83)	1.31 (1.29–1.32)
Hearing and language disability	4.75 (4.70–4.79)	1.78 (1.76–1.79)
Developmental disability	1.27 (1.25–1.29)	1.52 (1.49–1.54)
Mental disability	1.98 (1.93–2.03)	1.40 (1.37–1.44)
Internal organ disability	4.81 (4.74–4.88)	1.27 (1.25–1.29)
Female		
Status of disability		
With no disability	1.00	1.00
With disability	3.21 (3.20–3.22)	1.38 (1.37–1.38)
With disability (excluding respiratory disability)	3.19 (3.17–3.20)	1.37 (1.36–1.37)
Severity of disability (excluding respiratory disability)		
With no disability	1.00	1.00
Mild disability	3.58 (3.56–3.60)	1.41 (1.40–1.42)
Severe disability	2.58 (2.56–2.59)	1.29 (1.28–1.30)
Type of disability (excluding respiratory disability)		
With no disability	1.00	1.00
Physical disability	3.60 (3.58–3.62)	1.46 (1.45–1.47)
Brain injury	3.59 (3.55–3.64)	1.18 (1.17–1.20)
Facial disability	1.90 (1.66–2.18)	1.30 (1.13–1.50)
Visual disability	2.79 (2.75–2.82)	1.21 (1.19–1.23)
Hearing and language disability	3.58 (3.54–3.61)	1.40 (1.39–1.42)
Developmental disability	1.20 (1.18–1.22)	1.33 (1.31–1.36)
Mental disability	1.48 (1.45–1.52)	1.08 (1.05–1.11)
Internal organ disability	3.94 (3.88–4.01)	1.31 (1.29–1.34)

(B) Age	Crude OR (95% CI)	Adjusted OR (95% CI)^b^
Age < 60 years		
Status of disability		
With no disability	1.00	1.00
With disability	2.08 (2.07–2.09)	1.50 (1.50–1.51)
With disability (excluding respiratory disability)	2.05 (2.04–2.06)	1.48 (1.48–1.49)
Severity of disability (excluding respiratory disability)		
With no disability	1.00	1.00
Mild disability	1.96 (1.95–1.98)	1.39 (1.38–1.40)
Severe disability	2.14 (2.12–2.16)	1.59 (1.58–1.61)
Type of disability (excluding respiratory disability)		
With no disability	1.00	1.00
Physical disability	1.96 (1.95–1.97)	1.43 (1.42–1.45)
Brain injury	3.15 (3.10–3.20)	1.70 (1.67–1.73)
Facial disability	1.67 (1.47–1.91)	1.40 (1.22–1.61)
Visual disability	1.65 (1.62–1.68)	1.25 (1.22–1.27)
Hearing and language disability	1.95 (1.92–1.99)	1.50 (1.47–1.53)
Developmental disability	1.58 (1.56–1.60)	1.56 (1.54–1.58)
Mental disability	2.12 (2.08–2.17)	1.55 (1.52–1.58)
Internal organ disability	4.19 (4.12–4.27)	1.65 (1.62–1.68)
Age ≥ 60 years		
Status of disability		
With no disability	1.00	1.00
With disability	1.71 (1.70–1.71)	1.28 (1.28–1.29)
With disability (excluding respiratory disability)	1.68 (1.68–1.69)	1.26 (1.26–1.27)
Severity of disability (excluding respiratory disability)		
With no disability	1.00	1.00
Mild disability	1.67 (1.67–1.68)	1.28 (1.28–1.29)
Severe disability	1.70 (1.69–1.71)	1.22 (1.21–1.22)
Type of disability (excluding respiratory disability)		
With no disability	1.00	1.00
Physical disability	1.66 (1.66–1.67)	1.34 (1.33–1.35)
Brain injury	1.75 (1.74–1.77)	1.06 (1.05–1.07)
Facial disability	1.28 (1.12–1.47)	1.31 (1.14–1.52)
Visual disability	1.46 (1.44–1.47)	1.14 (1.12–1.15)
Hearing and language disability	1.90 (1.89–1.92)	1.32 (1.30–1.33)
Developmental disability	1.02 (0.98–1.05)	1.10 (1.07–1.14)
Mental disability	0.93 (0.91–0.96)	1.09 (1.06–1.12)
Internal organ disability	2.11 (2.08–2.14)	1.19 (1.17–1.21)

*OR* odds ratio, *CI* confidence interval.

^a^Adjusted for age (continuous), income level, place of residence, and Charlson cormorbidity index.

^b^Adjusted for sex, income level, place of residence, and Charlson cormorbidity index.

In the disability severity subgroup analysis after excluding people with respiratory disability, men with severe (aOR 1.48, 95% CI 1.47–1.49) and mild (aOR 1.40, 95% CI 1.39–1.41) disabilities had higher odds of COPD than those without disabilities. Similar patterns were found in women with severe (aOR 1.29, 95% CI 1.28–1.30) and mild (aOR 1.41, 95% CI 1.40–1.42) disabilities. Furthermore, all disability type subgroups had higher odds (range 1.08 to 1.78) of COPD compared to the no-disability subgroup in both men and women. The results of the multivariable analysis stratified by age were also similar. Hearing and language disability had the highest odds in the men (aOR 1.78, 95% CI 1.76–1.79) and younger people (aOR 1.78, 95% CI 1.76–1.79). Physical disability had the highest odds in the women (aOR 1.46, 95% CI 1.45–1.47) and older people (aOR 1.34, 95% CI 1.33–1.35).

## Discussion

To our knowledge, this is the first study to report that people with disabilities had a higher prevalence of COPD, which were also confirmed by the further analysis stratified by sex and age. We also identified several types of disabilities that are closely associated with COPD. Physical disability and hearing and language disability were associated with much higher prevalence of COPD, which suggests that regular screening should be provided for these individuals. Because earlier intervention can prevent lung function decline, many experts have stressed the importance of early diagnosis of COPD and concerted efforts have been made to promote spirometry screening for patients with COPD risk factors and symptoms^16^. Our findings provide important evidence of the need for public health interventions, such as screening programs for COPD, for people with disabilities.

Of the many risk factors for COPD, long-term cigarette smoking is the most important. Previous studies have provided inconsistent findings on smoking among people with disabilities. According to recent large nationwide surveys conducted in the United States^17^ and United Kingdom^18^, adults with disabilities are more likely to smoke compared to those without disabilities. A study using data from the 2013 Korea National Health and Nutrition Examination Survey reported that smoking rates were similar between adults with (21.4%) and without disabilities (21.5%)^19^. However, this Korean study did not evaluate whether people with disabilities were more likely to be heavy smokers than those without disabilities, including the smoking duration, number of cigarettes smoked daily, and pack-years. These findings suggest that differences in smoking behavior between people with and without disabilities might not be the only explanation for the high COPD prevalence seen in our study. For example, people with a disability are prone to diminished lung function^20^ and arrested lung development^21^, which might make them more vulnerable to lung damage due to cigarette smoking. In addition, people with disabilities might be more exposed to secondhand smoke throughout the life course^22,23^. Further studies of the effects of cigarette smoke exposure on lung function and chronic lung disease among people with disabilities are required.

Another important risk factor for COPD is exposure to indoor and outdoor air pollution, which is often problematic among people with disabilities. Poor housing ventilation is also associated with the development of COPD. Vulnerable populations, such as the elderly and those with cognitive and physical disabilities, are more likely to be affected by common hazards in the home, because they spend more time indoors^24^. A secondary analysis of a longitudinal birth cohort study in the United Kingdom found that levels of exposure to outdoor air pollution among children with an intellectual disability were significantly higher compared to those without an intellectual disability^25^, which might be attributable to residence in a socioeconomically deprived area with high levels of air pollution. According to a cross-sectional survey conducted in Canada, workers with disabilities are more exposed to occupational hazards than those without disabilities^26^, which might increase the risk of COPD. Improvements in working and housing environments can improve lung health and reduce COPD incidence^27,28^.

People with disabilities are more likely to be physically inactive^29^. This sedentary behavior is an important and highly prevalent risk factor for chronic diseases, including COPD^30^. Patients with COPD, who often complain of severe breathlessness, often limit their daily physical activity to minimize dyspnea; this inactivity can lead to muscle atrophy and deconditioning, consequently lowering activity tolerance. This vicious cycle may be exacerbated among people with disabilities. In addition, lower levels of physical activity are associated with a higher risk of exacerbation and exacerbation-related hospitalization, and also increase the risk of all-cause mortality in patients with COPD^31^. People with disabilities suffering from COPD may have a poorer prognosis than the general population. In people with disabilities, one or more physical attributes might be affected, which limits access to sport, fitness, and job- and household-related physical activity. It is necessary to promote an inclusive approach with respect to community programs and recreational, leisure, and sports activities^32^. For example, physical education in schools should be improved for all children by considering programs that are appropriate for children with disabilities.

Respiratory infection is an environmental risk factor for COPD and plays an important role in its pathogenesis and progression^33^. The nature of some disabilities may increase the risk of respiratory infection, which is one of the most common causes of mortality and healthcare utilization among people with intellectual disabilities^34^. Children with neurocognitive impairment often present with chronic or recurrent respiratory problems^35^, which might lead to reduced lung function and more severe respiratory symptoms in adulthood. Lower lung function in early adulthood with subsequent functional decline is as import as rapid lung function decline in normal-sized lungs with respect to the development of COPD^3^. A past history of tuberculosis is another important COPD risk factor in low- and middle-income countries^36^. Because of the overlap between tuberculosis and disability, people with disabilities are at greater risk of developing tuberculosis^37^, which in turn increases the risk of developing COPD.

One of the strengths of this study was the use of large-scale, real-world data based on a national health insurance claims database in Korea. No previous study has attempted to evaluate long-term trends in COPD prevalence among people with disabilities, or provided a detailed analysis according to the grade and type of disability. In this sense, our study has added new evidence to contribute to public health policy and practice, including the detection of health inequalities and the identification of priorities for early intervention. However, there were also some study limitations. First, our results cannot be extrapolated to other healthcare systems. Second, some clinical and demographic variables that may influence COPD development were not available from the NHID. In particular, because information on smoking status could only be obtained from individuals who participated in the national health checkup in the current or preceding year, it was not possible to confirm the current smoking status of more than half of the subjects based on the 2017 data. Finally, due to the cross-sectional nature of the study design, the causal relationships among variables could not be determined. Thus, further longitudinal studies are needed to verify our findings and gain insight into the mechanisms underlying these relationships.

## Conclusion

In conclusion, our study revealed a higher prevalence of COPD among people with than without disabilities. Respiratory system disease is a particularly important physical health issue in people with disabilities. Health problems caused by a combination of COPD and disability can impose a huge social and economic burden. The various barriers that the disabled encounter when seeking health care, and the difficulty of adhering to COPD treatment, must be overcome by developing appropriate public health interventions.

### Supplementary Information

Supplementary Tables.

## Data Availability

Data cannot be shared publicly because they belong to the National Health Insurance Service (NHIS). To request data from NHIS, researchers have to apply during the recruitment period and submit a research proposal. Raw data was available to researchers upon reasonable academic request and with the permission of the Korean NHIS Institutional Data Access (URL: https://nhiss.nhis.or.kr/bd/ab/bdaba000eng.do, Department of big data management: + 82-33-736-3481).
