# Supplementary material for: Growing disparity in the prevalence of chronic obstructive pulmonary disease between people with and without disabilities: a Korean nationwide serial cross-sectional study
Source: Sci Rep. 2023 Aug 14;13:13205. doi: 10.1038/s41598-023-39319-8 (PMC10425333; doi:10.1038/s41598-023-39319-8)
Supplement: Supplementary file 1 — Supplementary Tables. [file 41598_2023_39319_MOESM1_ESM.docx]

**Supplemental table 1**. Baseline characteristics of all participants between 2008 and 2017

|  | **2008** |  | **2009** |  | **2010** |  | **2011** |  | **2012** |  |
| --- | --- | --- | --- | --- | --- | --- | --- | --- | --- | --- |
|  | N | (Col%) | N | (Col%) | N | (Col%) | N | (Col%) | N | (Col%) |
| Total population | 50,364,957 |  | 50,636,578 |  | 50,941,054 |  | 51,269,500 |  | 51,565,237 |  |
| Status of disability |  |  |  |  |  |  |  |  |  |  |
| Without disability | 48,026,423 | (95.4) | 48,131,818 | (95.1) | 48,357,531 | (94.9) | 48,667,145 | (94.9) | 48,967,917 | (95.0) |
| With disability | 2,338,534 | (4.6) | 2,504,760 | (5.0) | 2,583,523 | (5.1) | 2,602,355 | (5.1) | 2,597,320 | (5.0) |
| Gender |  |  |  |  |  |  |  |  |  |  |
| Male | 25,300,799 | (50.2) | 25,423,272 | (50.2) | 25,571,691 | (50.2) | 25,733,659 | (50.2) | 25,871,561 | (50.2) |
| Female | 25,064,158 | (49.8) | 25,213,306 | (49.8) | 25,369,363 | (49.8) | 25,535,841 | (49.8) | 25,693,676 | (49.8) |
| Age (years) |  |  |  |  |  |  |  |  |  |  |
| Mean ± SD | 36.7 ± 20.4 |  | 37.2 ± 20.4 |  | 37.6 ± 20.6 |  | 38.1 ± 20.7 |  | 38.6 ± 20.8 |  |
| < 20 | 11,974,989 | (23.8) | 11,789,594 | (23.3) | 11,622,599 | (22.8) | 11,396,758 | (22.2) | 11,159,636 | (21.6) |
| 20–29 | 7,422,414 | (14.7) | 7,227,596 | (14.3) | 7,041,589 | (13.8) | 6,928,955 | (13.5) | 6,842,113 | (13.3) |
| 30–39 | 8,655,067 | (17.2) | 8,564,567 | (16.9) | 8,489,820 | (16.7) | 8,401,961 | (16.4) | 8,331,031 | (16.2) |
| 40–49 | 8,779,206 | (17.4) | 8,850,346 | (17.5) | 8,842,772 | (17.4) | 8,873,850 | (17.3) | 8,879,155 | (17.2) |
| 50–59 | 6,224,943 | (12.4) | 6,601,044 | (13.0) | 7,055,188 | (13.9) | 7,531,027 | (14.7) | 7,812,692 | (15.2) |
| 60–69 | 4,021,437 | (8.0) | 4,105,831 | (8.1) | 4,200,860 | (8.3) | 4,230,293 | (8.3) | 4,346,616 | (8.4) |
| 70–79 | 2,397,641 | (4.8) | 2,543,169 | (5.0) | 2,665,172 | (5.2) | 2,818,482 | (5.5) | 3,020,960 | (5.9) |
| ≥ 80 | 889,260 | (1.8) | 954,431 | (1.9) | 1,023,054 | (2.0) | 1,088,174 | (2.1) | 1,173,034 | (2.3) |
| Income level |  |  |  |  |  |  |  |  |  |  |
| First quartile^a^ | 9,986,217 | (19.8) | 10,180,664 | (20.1) | 10,224,050 | (20.1) | 10,334,566 | (20.2) | 10,404,061 | (20.2) |
| Second quartile | 10,287,963 | (20.4) | 10,343,713 | (20.4) | 10,268,133 | (20.2) | 10,318,775 | (20.1) | 10,340,991 | (20.1) |
| Third quartile | 13,082,725 | (26.0) | 13,062,482 | (25.8) | 13,134,678 | (25.8) | 13,065,461 | (25.5) | 13,106,951 | (25.4) |
| Fourth quartile | 16,015,756 | (31.8) | 16,097,101 | (31.8) | 16,282,073 | (32.0) | 16,464,340 | (32.1) | 16,649,329 | (32.3) |
| Unknown | 992,296 | (2.0) | 952,618 | (1.9) | 1,032,120 | (2.0) | 1,086,358 | (2.1) | 1,063,905 | (2.1) |
| Place of residence |  |  |  |  |  |  |  |  |  |  |
| Metropolitan | 30,736,624 | (61.0) | 31,318,626 | (61.9) | 31,405,467 | (61.7) | 32,549,886 | (63.5) | 32,640,567 | (63.3) |
| Urban | 14,681,547 | (29.2) | 14,391,083 | (28.4) | 14,586,189 | (28.6) | 13,723,656 | (26.8) | 14,060,591 | (27.3) |
| Rural | 4,853,634 | (9.6) | 4,857,327 | (9.6) | 4,873,375 | (9.6) | 4,887,864 | (9.5) | 4,756,681 | (9.2) |
| Unknown | 93,152 | (0.2) | 69,542 | (0.1) | 76,023 | (0.2) | 108,094 | (0.2) | 107,398 | (0.2) |
| CCI |  |  |  |  |  |  |  |  |  |  |
| 0 | 33,448,991 | (66.4) | 32,507,148 | (64.2) | 32,352,208 | (63.5) | 32,171,900 | (62.8) | 31,619,612 | (61.3) |
| 1–2 | 13,559,245 | (26.9) | 14,555,852 | (28.7) | 14,879,050 | (29.2) | 15,347,269 | (29.9) | 15,980,164 | (31.0) |
| 3–4 | 2,406,012 | (4.8) | 2,560,206 | (5.1) | 2,656,312 | (5.2) | 2,707,212 | (5.3) | 2,857,023 | (5.5) |
| ≥ 5 | 950,709 | (1.9) | 1,013,372 | (2.0) | 1,053,484 | (2.1) | 1,043,119 | (2.0) | 1,108,438 | (2.1) |

Abbreviation: SD, Standard Deviation; CCI, Charlson Cormorbidity Index.

^a^ Medical Aid beneficiaries were merged into the first quartile group.

**Supplemental table 1** (continued)

|  | **2013** |  | **2014** |  | **2015** |  | **2016** |  | **2017** |  |
| --- | --- | --- | --- | --- | --- | --- | --- | --- | --- | --- |
|  | N | (Col%) | N | (Col%) | N | (Col%) | N | (Col%) | N | (Col%) |
| Total population | 51,783,064 |  | 52,073,737 |  | 52,359,705 |  | 52,585,429 |  | 52,712,239 |  |
| Status of disability |  |  |  |  |  |  |  |  |  |  |
| Without disability | 2,584,797 | (5.0) | 2,575,019 | (4.9) | 2,576,941 | (4.9) | 2,597,885 | (4.9) | 2,627,365 | (5.0) |
| With disability | 49,198,267 | (95.0) | 49,498,718 | (95.1) | 49,782,764 | (95.1) | 49,987,544 | (95.1) | 50,084,874 | (95.0) |
| Gender |  |  |  |  |  |  |  |  |  |  |
| Male | 25,961,391 | (50.1) | 26,100,680 | (50.1) | 26,241,734 | (50.1) | 26,341,211 | (50.1) | 26,390,827 | (50.1) |
| Female | 25,821,673 | (49.9) | 25,973,057 | (49.9) | 26,117,971 | (49.9) | 26,244,218 | (49.9) | 26,321,412 | (49.9) |
| Age (years) |  |  |  |  |  |  |  |  |  |  |
| Mean ± SD | 39.1 ± 20.9 |  | 39.5 ± 21.0 |  | 40.0 ± 21.1 |  | 40.5 ± 21.2 |  | 41.0 ± 21.3 |  |
| < 20 | 10,892,576 | (21.0) | 10,625,940 | (20.4) | 10,367,987 | (19.8) | 10,103,912 | (19.2) | 9,800,667 | (18.6) |
| 20–29 | 6,813,447 | (13.2) | 6,877,242 | (13.2) | 6,942,171 | (13.3) | 7,002,580 | (13.3) | 7,039,984 | (13.4) |
| 30–39 | 8,183,690 | (15.8) | 8,006,175 | (15.4) | 7,894,914 | (15.1) | 7,780,066 | (14.8) | 7,634,258 | (14.5) |
| 40–49 | 8,962,008 | (17.3) | 8,991,256 | (17.3) | 8,930,341 | (17.1) | 8,874,023 | (16.9) | 8,788,690 | (16.7) |
| 50–59 | 8,057,652 | (15.6) | 8,276,374 | (15.9) | 8,401,320 | (16.1) | 8,510,941 | (16.2) | 8,583,409 | (16.3) |
| 60–69 | 4,495,473 | (8.7) | 4,740,420 | (9.1) | 5,117,331 | (9.8) | 5,431,093 | (10.3) | 5,723,391 | (10.9) |
| 70–79 | 3,122,433 | (6.0) | 3,195,012 | (6.1) | 3,223,706 | (6.2) | 3,284,451 | (6.3) | 3,423,916 | (6.5) |
| ≥ 80 | 1,255,785 | (2.4) | 1,361,318 | (2.6) | 1,481,935 | (2.8) | 1,598,363 | (3.0) | 1,717,924 | (3.3) |
| Income level |  |  |  |  |  |  |  |  |  |  |
| First quartile^a^ | 10,373,723 | (20.0) | 10,394,534 | (20.0) | 10,464,158 | (20.0) | 10,613,144 | (20.2) | 10,654,182 | (20.2) |
| Second quartile | 10,411,072 | (20.1) | 10,442,021 | (20.1) | 10,463,697 | (20.0) | 10,436,389 | (19.9) | 10,426,396 | (19.8) |
| Third quartile | 13,105,452 | (25.3) | 13,188,156 | (25.3) | 13,211,306 | (25.2) | 13,193,127 | (25.1) | 13,156,971 | (25.0) |
| Fourth quartile | 16,768,204 | (32.4) | 16,882,776 | (32.4) | 17,041,817 | (32.6) | 17,146,199 | (32.6) | 17,260,428 | (32.7) |
| Unknown | 1,124,613 | (2.2) | 1,166,250 | (2.2) | 1,178,727 | (2.3) | 1,196,570 | (2.3) | 1,214,262 | (2.3) |
| Place of residence |  |  |  |  |  |  |  |  |  |  |
| Metropolitan | 32,713,285 | (63.2) | 32,830,126 | (63.1) | 33,075,098 | (63.2) | 33,012,217 | (62.8) | 32,887,047 | (62.4) |
| Urban | 14,297,885 | (27.6) | 14,568,642 | (28.0) | 14,778,203 | (28.2) | 15,042,201 | (28.6) | 15,279,473 | (29.0) |
| Rural | 4,673,653 | (9.0) | 4,592,509 | (8.8) | 4,465,020 | (8.5) | 4,498,318 | (8.6) | 4,519,089 | (8.6) |
| Unknown | 98,241 | (0.2) | 82,460 | (0.2) | 41,384 | (0.1) | 32,693 | (0.1) | 26,630 | (0.1) |
| CCI |  |  |  |  |  |  |  |  |  |  |
| 0 | 31,731,959 | (61.3) | 31,168,797 | (59.9) | 31,220,816 | (59.6) | 30,207,225 | (57.4) | 30,346,892 | (57.6) |
| 1–2 | 15,914,468 | (30.7) | 16,582,109 | (31.8) | 16,642,711 | (31.8) | 17,551,310 | (33.4) | 17,325,153 | (32.9) |
| 3–4 | 2,970,991 | (5.7) | 3,098,129 | (5.9) | 3,199,339 | (6.1) | 3,416,518 | (6.5) | 3,550,426 | (6.7) |
| ≥ 5 | 1,165,646 | (2.3) | 1,224,702 | (2.4) | 1,296,839 | (2.5) | 1,410,376 | (2.7) | 1,489,768 | (2.8) |

Abbreviation: SD, Standard Deviation; CCI, Charlson Cormorbidity Index.

^a^ Medical Aid beneficiaries were merged into the first quartile group.

**Supplemental table 2.** Severity and type of disability among people with disabilities between 2008 and 2017

|  | **2008** |  | **2009** |  | **2010** |  | **2011** |  | **2012** |  |
| --- | --- | --- | --- | --- | --- | --- | --- | --- | --- | --- |
|  | N | (Col%) | N | (Col%) | N | (Col%) | N | (Col%) | N | (Col%) |
| All | 2,338,534 |  | 2,504,760 |  | 2,583,523 |  | 2,602,355 |  | 2,597,320 |  |
| Severity of disability |  |  |  |  |  |  |  |  |  |  |
| Mild disability | 1,343,375 | (57.5) | 1,462,048 | (58.4) | 1,525,649 | (59.1) | 1,552,604 | (59.7) | 1,563,006 | (60.2) |
| Severe disability | 995,159 | (42.6) | 1,042,712 | (41.6) | 1,057,874 | (41.0) | 1,049,751 | (40.3) | 1,034,314 | (39.8) |
| Type of disability |  |  |  |  |  |  |  |  |  |  |
| Physical disability | 1,262,690 | (54.0) | 1,354,031 | (54.1) | 1,391,321 | (53.9) | 1,393,146 | (53.5) | 1,382,442 | (53.2) |
| Brain injury | 234,522 | (10.0) | 252,928 | (10.1) | 259,714 | (10.1) | 260,924 | (10.0) | 261,159 | (10.1) |
| Facial disability | 2,282 | (0.1) | 2,436 | (0.1) | 2,904 | (0.1) | 3,046 | (0.1) | 2,997 | (0.1) |
| Visual disability | 236,712 | (10.1) | 248,169 | (9.9) | 254,797 | (9.9) | 256,750 | (9.9) | 258,055 | (9.9) |
| Hearing and language disability | 245,054 | (10.5) | 267,598 | (10.7) | 282,636 | (10.9) | 287,084 | (11.0) | 285,282 | (11.0) |
| Developmental disability^a^ | 167,408 | (7.2) | 174,975 | (7.0) | 180,458 | (7.0) | 186,228 | (7.2) | 192,429 | (7.4) |
| Mental disability | 75,690 | (3.2) | 83,635 | (3.3) | 87,300 | (3.4) | 89,077 | (3.4) | 88,551 | (3.4) |
| Internal organ disability^b^ | 98,080 | (4.2) | 104,131 | (4.2) | 107,808 | (4.2) | 110,222 | (4.2) | 111,211 | (4.3) |
| Respiratory problems | 16,096 | (0.7) | 16,857 | (0.7) | 16,585 | (0.6) | 15,878 | (0.6) | 15,194 | (0.6) |

^a^ Developmental disability included intellectual disability and autism.

^b^ Internal organ disability included renal failure, heart problems, liver disease, ostomy, and epilepsy.

**Supplemental table 2** (continued)

|  | **2013** |  | **2014** |  | **2015** |  | **2016** |  | **2017** |  |
| --- | --- | --- | --- | --- | --- | --- | --- | --- | --- | --- |
|  | N | (Col%) | N | (Col%) | N | (Col%) | N | (Col%) | N | (Col%) |
| All | 2,584,797 |  | 2,575,019 |  | 2,576,941 |  | 2,597,885 |  | 2,627,365 |  |
| Severity of disability |  |  |  |  |  |  |  |  |  |  |
| Mild disability | 1,564,483 | (60.5) | 1,564,186 | (60.7) | 1,571,092 | (61.0) | 1,591,533 | (61.3) | 1,618,856 | (61.6) |
| Severe disability | 1,020,314 | (39.5) | 1,010,833 | (39.3) | 1,005,849 | (39.0) | 1,006,352 | (38.7) | 1,008,509 | (38.4) |
| Type of disability |  |  |  |  |  |  |  |  |  |  |
| Physical disability | 1,368,562 | (53.0) | 1,354,964 | (52.6) | 1,345,028 | (52.2) | 1,332,875 | (51.3) | 1,319,712 | (50.2) |
| Brain injury | 258,445 | (10.0) | 256,050 | (9.9) | 257,380 | (10.0) | 257,987 | (9.9) | 258,610 | (9.8) |
| Facial disability | 2,951 | (0.1) | 2,928 | (0.1) | 2,749 | (0.1) | 2,724 | (0.1) | 2,725 | (0.1) |
| Visual disability | 258,259 | (10.0) | 257,866 | (10.0) | 258,775 | (10.0) | 259,105 | (10.0) | 259,423 | (9.9) |
| Hearing and language disability | 281,954 | (10.9) | 279,517 | (10.9) | 278,321 | (10.8) | 298,130 | (11.5) | 327,694 | (12.5) |
| Developmental disability^a^ | 198,685 | (7.7) | 205,051 | (8.0) | 212,497 | (8.3) | 219,601 | (8.5) | 226,281 | (8.6) |
| Mental disability | 88,732 | (3.4) | 89,089 | (3.5) | 89,965 | (3.5) | 90,898 | (3.5) | 91,560 | (3.5) |
| Internal organ disability^b^ | 112,815 | (4.4) | 115,894 | (4.5) | 118,955 | (4.6) | 123,563 | (4.8) | 128,565 | (4.9) |
| Respiratory problems | 14,394 | (0.6) | 13,660 | (0.5) | 13,271 | (0.5) | 13,002 | (0.5) | 12,795 | (0.5) |

^a^ Developmental disability included intellectual disability and autism.

^b^ Internal organ disability included renal failure, heart problems, liver disease, ostomy, and epilepsy.

**Supplemental table 3.** Prevalence of chronic obstructive pulmonary disease among people with and without disabilities stratified by gender between 2008 and 2017

|  | Year | **Without disability** | | **Without disability** | | *P* value^a^ | **Without disability**  **(excluding respiratory problems)** | | *P* value^a^ |
| --- | --- | --- | --- | --- | --- | --- | --- | --- | --- |
|  |  | No. of COPD cases | Crude prevalence rate, % | No. of COPD cases | Crude prevalence rate, % |  | No. of COPD cases | Crude prevalence rate, % |  |
| **All** | 2008 | 2,077,523 | 4.3 | 309,219 | 13.2 | <0.001 | 295,920 | 12.7 | <0.001 |
|  | 2009 | 2,611,378 | 5.4 | 402,072 | 16.1 | <0.001 | 387,672 | 15.6 | <0.001 |
|  | 2010 | 3,094,052 | 6.4 | 473,839 | 18.3 | <0.001 | 459,385 | 17.9 | <0.001 |
|  | 2011 | 3,571,362 | 7.3 | 530,743 | 20.4 | <0.001 | 516,717 | 20.0 | <0.001 |
|  | 2012 | 4,093,604 | 8.4 | 585,908 | 22.6 | <0.001 | 572,259 | 22.2 | <0.001 |
|  | 2013 | 4,515,275 | 9.2 | 625,875 | 24.2 | <0.001 | 612,810 | 23.8 | <0.001 |
|  | 2014 | 4,950,764 | 10.0 | 663,786 | 25.8 | <0.001 | 651,269 | 25.4 | <0.001 |
|  | 2015 | 5,358,447 | 10.8 | 703,598 | 27.3 | <0.001 | 691,301 | 27.0 | <0.001 |
|  | 2016 | 5,832,683 | 11.7 | 752,014 | 29.0 | <0.001 | 739,885 | 28.6 | <0.001 |
|  | 2017 | 6,282,610 | 12.5 | 802,669 | 30.6 | <0.001 | 790,671 | 30.2 | <0.001 |
|  |  |  |  |  |  |  |  |  |  |
| **Male** | 2008 | 915,768 | 3.8 | 173,785 | 12.4 | <0.001 | 163,351 | 11.7 | <0.001 |
|  | 2009 | 1,140,808 | 4.8 | 218,432 | 14.8 | <0.001 | 207,232 | 14.1 | <0.001 |
|  | 2010 | 1,348,000 | 5.6 | 253,463 | 16.8 | <0.001 | 242,299 | 16.2 | <0.001 |
|  | 2011 | 1,552,769 | 6.4 | 281,998 | 18.6 | <0.001 | 271,192 | 18.0 | <0.001 |
|  | 2012 | 1,777,479 | 7.3 | 310,050 | 20.5 | <0.001 | 299,581 | 19.9 | <0.001 |
|  | 2013 | 1,959,782 | 8.0 | 330,203 | 21.9 | <0.001 | 320,249 | 21.4 | <0.001 |
|  | 2014 | 2,147,847 | 8.7 | 349,592 | 23.3 | <0.001 | 340,120 | 22.9 | <0.001 |
|  | 2015 | 2,322,679 | 9.4 | 370,251 | 24.7 | <0.001 | 360,971 | 24.2 | <0.001 |
|  | 2016 | 2,528,511 | 10.2 | 395,575 | 26.2 | <0.001 | 386,485 | 25.8 | <0.001 |
|  | 2017 | 2,722,083 | 11 | 421,795 | 27.7 | <0.001 | 412,802 | 27.3 | <0.001 |
|  |  |  |  |  |  |  |  |  |  |
| **Female** | 2008 | 1,161,755 | 4.8 | 135,434 | 14.5 | <0.001 | 132,569 | 14.3 | <0.001 |
|  | 2009 | 1,470,570 | 6.1 | 183,640 | 17.9 | <0.001 | 180,440 | 17.7 | <0.001 |
|  | 2010 | 1,746,052 | 7.2 | 220,376 | 20.6 | <0.001 | 217,086 | 20.3 | <0.001 |
|  | 2011 | 2,018,593 | 8.3 | 248,745 | 23.0 | <0.001 | 245,525 | 22.8 | <0.001 |
|  | 2012 | 2,316,125 | 9.4 | 275,858 | 25.5 | <0.001 | 272,678 | 25.3 | <0.001 |
|  | 2013 | 2,555,493 | 10.3 | 295,672 | 27.4 | <0.001 | 292,561 | 27.2 | <0.001 |
|  | 2014 | 2,802,917 | 11.3 | 314,194 | 29.2 | <0.001 | 311,149 | 29.0 | <0.001 |
|  | 2015 | 3,035,768 | 12.1 | 333,347 | 30.9 | <0.001 | 330,330 | 30.8 | <0.001 |
|  | 2016 | 3,304,172 | 13.1 | 356,439 | 32.8 | <0.001 | 353,400 | 32.6 | <0.001 |
|  | 2017 | 3,560,527 | 14.1 | 380,874 | 34.5 | <0.001 | 377,869 | 34.4 | <0.001 |

^a^ Statistical significance of the difference compared to people without disability.
